# Appearances can be deceptive: acute myeloid leukaemia simulating chronic lymphocytic leukaemia

**DOI:** 10.1515/almed-2025-0184

**Published:** 2026-05-20

**Authors:** Beatriz Gómez Horsefield, David Cruz García, Ana Garzó Moreno, Esperanza Tuset Andujar, Leire Saiz Sierra, Maite Serrando Querol

**Affiliations:** Institut Català Oncologia, Girona, Spain; Laboratori Territorial ICS-IAS Girona, Hospital Universitari Doctor Josep Trueta, Girona, Spain; Comisión de Hematología de la Sociedad Española de Medicina de Laboratorio (SEMEDLAB), Girona, Spain; Research Group of Clinical Anatomy, Embryology and Neuroscience (NEOMA), Department of Medical Sciences, University of Girona, Girona, Spain

**Keywords:** immunophenotype, cell morphology, peripheral blood feautures

## Abstract

**Objectives:**

To report a rare case of acute myeloid leukemia (AML) with abnormal chromatin clumping (ACC) in myeloblasts mimicking chronic lymphocytic leukemia (CLL), highlighting the associated diagnostic challenges.

**Case presentation:**

An 82-year-old woman presented with fatigue, weight loss, and pancytopenia. Peripheral blood film revealed 90 % small mononuclear cells with clumped chromatin and basket cells reminiscent of CLL. Flow cytometry of peripheral blood demonstrated a dim CD45-positive blast population negative for B-cell markers (CD19, CD20, CD10) and lacking light-chain restriction. Bone marrow aspiration confirmed hypercellularity with 94 % blasts expressing CD34, CD117, CD123, MPO, and aberrant CD7. Cytogenetic analysis revealed a complex karyotype with 46,XX, 1p36 abnormalities, and next-generation sequencing identified mutations in *EZH2, PHF6, WT1,* and *STAG2*. The final diagnosis was AML with myelodysplasia-related mutations (adverse risk, ELN-2022). Despite cytoreductive therapy, the patient showed no response and died within three weeks.

**Conclusions:**

This case highlights the diagnostic challenge posed by AML with ACC, a morphology that can closely resemble CLL. Recognition of this pattern is essential to avoid misclassification, as therapeutic strategies and prognosis differ significantly. Only three similar cases have been reported, underscoring the exceptional rarity and clinical importance of this presentation.

## Introduction

Acute myeloid leukemia (AML) is a clonal hematologic malignancy characterized by the proliferation of immature myeloid precursors, leading to bone marrow failure. Chronic lymphocytic leukemia (CLL), in contrast, is a mature B-cell neoplasm with an indolent course. Morphologic evaluation of blasts remains essential for the diagnosis of acute myeloid leukemia (AML) and continues to be a characteristic feature in the first step of the diagnostic workup, despite advances in immunophenotyping and molecular testing. Abnormal chromatin clumping (ACC) is a rare morphologic feature classically described in lymphoid neoplasms, particularly chronic lymphocytic leukemia. Although isolated cases of acute myeloid leukemia with abnormal chromatin condensation in blasts have been reported, these descriptions are limited to morphological observations and, to date, ACC in myeloid blasts has not been associated with a distinct clinicopathologic entity or a specific genetic or molecular alteration. [1], 2]. Its presence in myeloblasts is exceptionally uncommon and may cause diagnostic confusion, especially in elderly patients where CLL is more prevalent.

Recognizing this atypical morphology is critical because AML and CLL differ markedly in treatment and prognosis. AML usually requires intensive induction chemotherapy (e.g., cytarabine plus anthracycline), often followed by consolidation therapy or hematopoietic stem cell transplantation in eligible patients. In contrast, CLL management is often risk-adapted**,** ranging from watchful waiting in asymptomatic cases to targeted therapies such as BTK inhibitors (ibrutinib), BCL-2 inhibitors (venetoclax), or anti-CD20 monoclonal antibodies [3]. These differences underscore the importance of accurate diagnosis to avoid inappropriate therapy.

We describe an unusual and rare case of abnormal chromatin clumping (ACC) in myeloblasts in an elderly man with acute myeloid leukemia (AML) that mimicked chronic lymphocytic leukemia (CLL). Recognizing this atypical morphology is important as both neoplasms (AML and CLL) differ in their treatment and prognosis.

## Case presentation

An 82-year-old woman presented to the emergency department with progressive fatigue, anorexia, and unintentional weight loss over the past two to three weeks, which significantly affected her previous level of autonomy. Her family history was notable: her mother died at the age of 30 from an unknown cause, and her 53-year-old daughter died of squamous cell carcinoma of the cervix.

On admission, the complete blood count revealed hemoglobin of 69 g/L, platelet count of 29 × 10^9^/L, and a white blood cell count of 61.14 × 10^9^/L; manual review of the peripheral blood smear showed lymphocytes accounting for 91.3 % and blasts for 8.7 %. The white cell differential fluorescence scatterplot obtained by the Sysmex XN-series haematology analyser showed an abnormal cell population in the lymphoid/monocytic region represented as a grey cloud. Physical examination revealed no lymphadenopathy, hepatomegaly, or palpable masses. Biochemical analysis showed serum ferritin of 703 ng/mL (reference range 5–204 ng/mL), folic acid of 5 ng/mL (3.1–20 ng/mL), vitamin B12 of 412 pg/mL (187–883 pg/mL), and TSH of 1.52 μUI/mL (0.35–4.94 μUI/mL).

**Figure 1:**
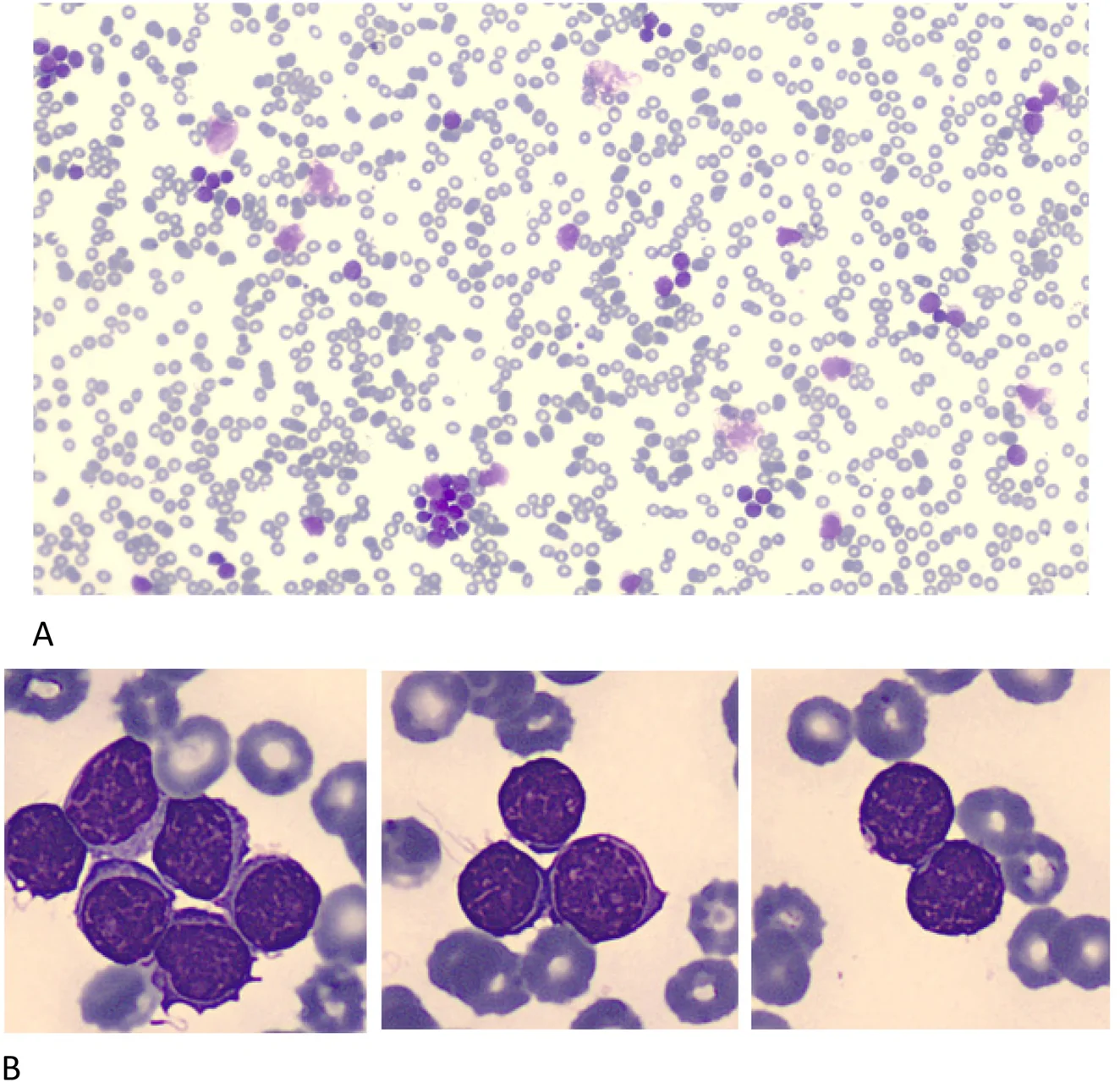
Peripheral blood film feautures (PBF). (A) PBF revealed 90 % small mononuclear cells with abundant basket cells reminding of Gumprecht shadows of chronic lymphocytic leukemia. (B) In more detail, there mononuclear cells showed abnormal nuclear chromatin simulating condensed chromatin of grumeleeé appearance, come with visible nucleoli. Image taken from CellaVision 9600.

Figure 1A and B shows peripheral blood film feautures. Cytology demonstrated approximately 90 % atypical small mononuclear cells with abnormal nuclear chromatin and visible nucleoli, along with abundant basket cells reminiscent of Gumprecht shadows typically seen in chronic lymphocytic leukemia. The few granulocytes present did not exhibit abnormal chromatin clumping, although some were polynuclear with pleukaryocyte morphology. No prior hematological records were available for this patient, and therefore the presence of persistent lymphocytosis suggestive of an underlying lymphoproliferative neoplasm could not be assessed.

**Figure 2:**
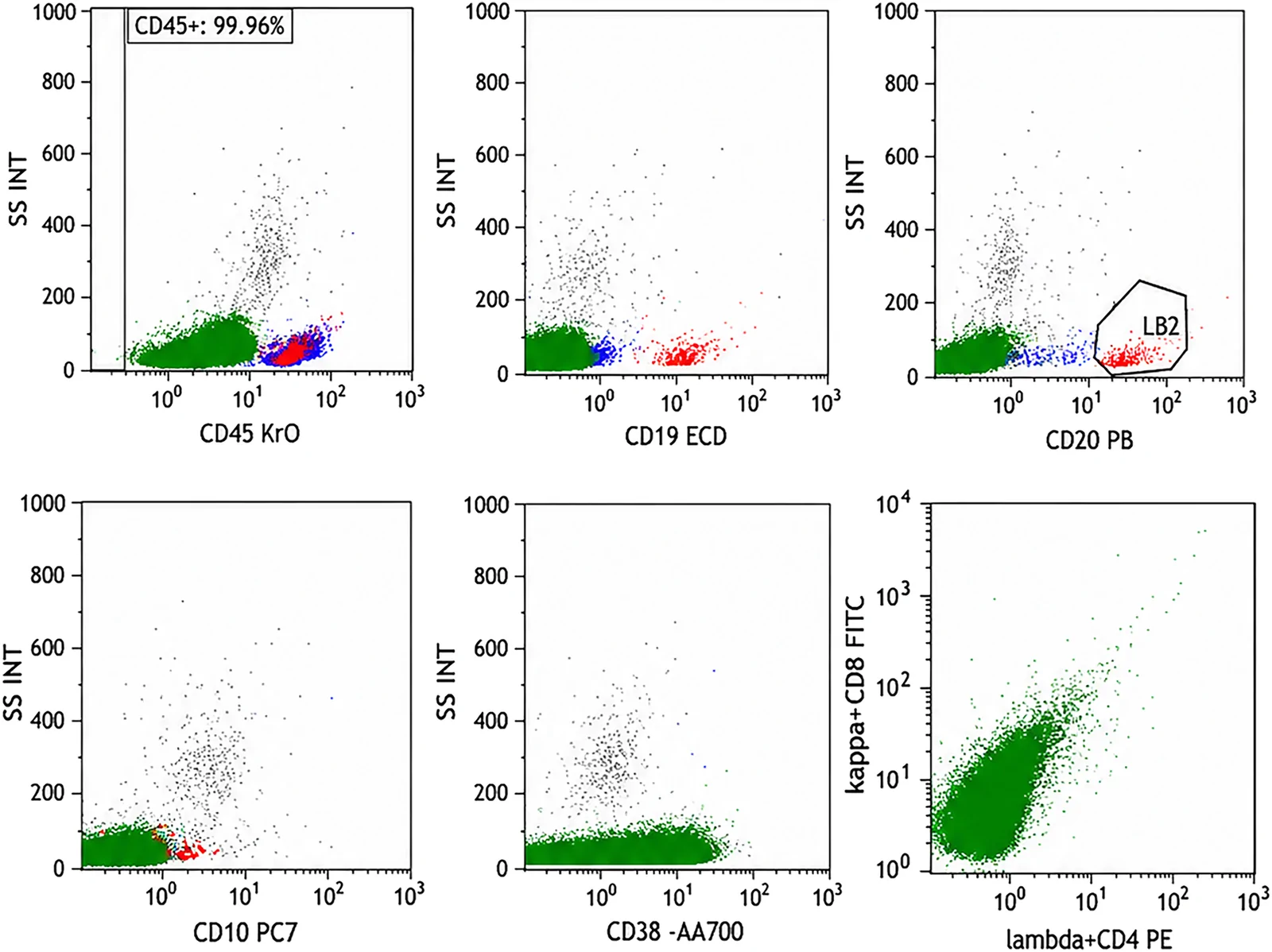
Immunophenotyping by flow cytometry in peripheral blood revealed a dim CD45 positive population comprising 99 % of all singlets shown in green being negative for CD19, CD20 and CD10, partially positive for CD38 and absence of clonality with a homogenous distribution of kappa/lambda chains.

Based on these findings, an initial lymphoproliferative syndrome screening was performed, with chronic lymphoproliferative syndrome considered the most likely diagnosis. However, flow cytometry revealed a dim CD45-positive population comprising 99 % of singlets, negative for CD19, CD20, and CD10, partially positive for CD38, and showing no evidence of clonality with a homogeneous distribution of kappa and lambda chains (Figure 2).

**Figure 3:**
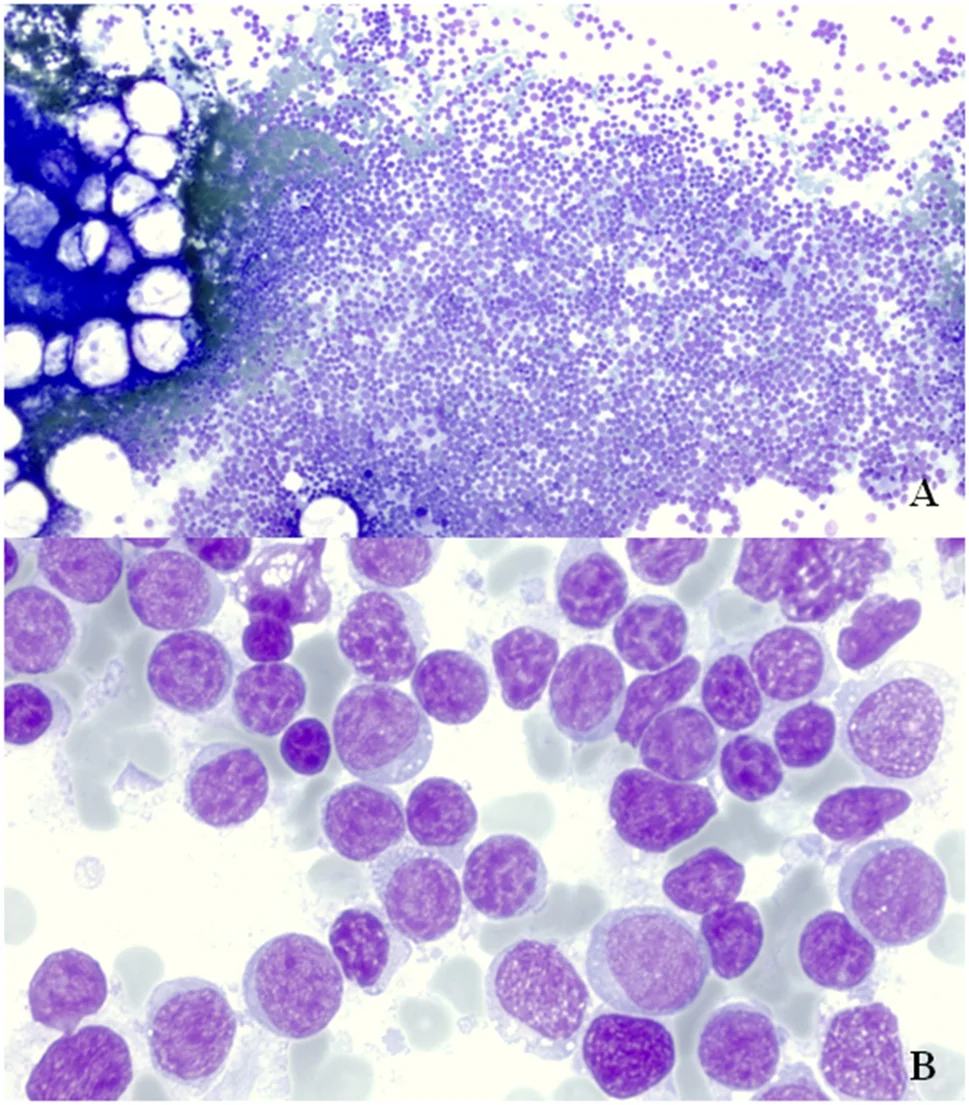
Bone marrow aspirate findings. (A) Bone marrow aspirate showing monomorphic hypercellularity with decreased fat cells.(B) Higher magnification image showing that the monomorphic hypercellularity was composed of medium-sized mononuclear cells with heterogeneous morphology and two cell populations: a predominant population with round nuclei, clumped chromatin, and scant cytoplasm, and a second population (11 %) of larger cells with immature chromatin.

A bone marrow aspirate was performed due to suspicion of acute leukemia, and the morphology mirrored the findings observed in peripheral blood (Figure 3A and B). No morphological abnormalities suggestive of dysplasia were identified in the granulocytic, erythroid, or megakaryocytic lineages.

Flow cytometry of the bone marrow, performed using an extended acute leukemia panel, identified a predominant blast population (94 %) characterized by low side scatter and weak CD45 expression. The blasts expressed immaturity-associated antigens including CD34 and CD123, with partial TdT expression (22 %), together with myeloid-lineage markers such as MPO (10 %), CD117, partial CD33, partial CD13 (10 %), and partial CD11b (14 %). HLA-DR, CD38, CD99, and partial CD56 were also expressed. Aberrant expression of lymphoid-associated antigens CD7 (85 %) and partial CD2 (12 %) was observed, while other lineage-defining markers were absent. According to WHO criteria, mixed-phenotype acute leukemia was excluded based on MPO positivity and absence of cytoplasmic or surface CD3 expression.

Cytogenetic analysis revealed a karyotype of 46,XX with adherent material at 1p36 and two marker chromosomes, consistent with a complex karyotype. Molecular testing for FLT3-ITD, FLT3-TKD, NPM1, and IDH1 was negative. Next-generation sequencing identified pathogenic variants in EZH2 (VAF 46.2 %), PHF6 (44.1 %), WT1 (42.2 %), and STAG2 (4.3 %).

Although abnormal chromatin clumping has historically been described in the context of myelodysplastic or dysgranulopoietic disorders, this case does not fulfil morphological criteria for myelodysplastic syndrome. Dysplasia was carefully assessed in granulocytic, erythroid, and megakaryocytic lineages and was not identified at any stage, in either peripheral blood or bone marrow. In contrast to previously described ACC-associated disorders, in which abnormal chromatin clumping predominantly affects mature granulocytes and is frequently accompanied by dysplasia, in this case ACC was strictly confined to myeloblasts, with complete sparing of mature neutrophils.

The patient was diagnosed according to ICC [4] and WHO 2022 [5] criteria as having acute myeloid leukemia with mutations related to myelodysplasia, classified as adverse risk according to ELN-2022 [6]. She received cytoreductive therapy without response, broad-spectrum antibiotics for a dental infection, and transfusion support. Ultimately, she was admitted for palliative care and died three weeks later.

## Discussion

Abnormal chromatin clumping (ACC) was originally described in the 1980s in the context of a rare myelodysplastic disorder affecting predominantly mature granulocytes, characterized by loss of nuclear segmentation, granulocytic hyperplasia, circulating immature granulocytes, and frequent dysplastic changes in erythroid and megakaryocytic lineages. This entity shows marked morphological overlap with acquired Pelger–Huët anomaly and has been most commonly associated with dysgranulopoietic conditions, including atypical chronic myeloid leukemia (aCML) [7], 8].

In contrast, this case does not fulfil morphological criteria for myelodysplastic syndrome. Dysplasia was systematically assessed in granulocytic, erythroid, and megakaryocytic lineages in both peripheral blood and bone marrow and was not identified at any stage. Mature neutrophils did not show abnormal chromatin clumping or other dysplastic features, and there was no evidence of dyserythropoiesis or dysmegakaryopoiesis. Therefore, the resemblance to previously described ACC-associated myelodysplastic or dysgranulopoietic disorders is limited exclusively to the presence of the chromatin abnormality, rather than to a shared clinicopathologic phenotype.

Since 2016, only three cases of acute myeloid leukemia (AML) with abnormal chromatin clumping in myeloblasts have been reported in the literature. In most of these cases, ACC occurred in association with dysplastic features or within a myelodysplastic or dysgranulopoietic context**.** In contrast, the present case demonstrates ACC strictly confined to myeloblasts**,** with complete sparing of mature granulocytes, representing a distinctive and previously underrecognized morphological presentation.

Despite the absence of morphological dysplasia, this case met genetic criteria for AML with myelodysplasia-related mutations according to WHO 2022 and ICC classifications, based on the presence of pathogenic variants in EZH2, PHF6, WT1, and STAG2 and a complex karyotype. This highlights the increasing importance of molecular findings in disease classification and illustrates a potential discordance between morphology and genetic background in AML.

From a diagnostic perspective, this case is particularly relevant because the condensed chromatin pattern in blasts resulted in a striking morphological mimicry of chronic lymphocytic leukemia (CLL). The abnormal cells were initially interpreted as lymphocytes, supported by automated scatterplot findings and the presence of basket cells, leading to an initial suspicion of a lymphoproliferative disorder. Recognition of ACC in blasts and correlation with immunophenotypic findings were essential to avoid misclassification.

In summary, this case expands the morphological spectrum of AML by demonstrating that abnormal chromatin clumping may occur exclusively in myeloblasts, in the absence of dysplasia, while closely simulating CLL. Awareness of this rare presentation is crucial to prevent diagnostic errors with significant therapeutic and prognostic implications.
